# Discovering the underlying typology of emergency departments

**DOI:** 10.1186/s12874-021-01305-x

**Published:** 2021-06-05

**Authors:** Marine Demarquet, Laurie Fraticelli, Julie Freyssenge, Clément Claustre, Mikaël Martinez, Jonathan Duchenne, Carlos El Khoury, Abdesslam Redjaline, Karim Tazarourte

**Affiliations:** 1 Centre Hospitalier de Fleyriat, Bourg-en-Bresse, France; 2 Observatoire Régional Des Urgences (ORU), Agence Régionale de Santé Auvergne Rhône-Alpes, Lyon, France; 3 RESCUe-RESUVal Network, Hospital Center Lucien Hussel, Montée Docteur Maurice Chapuis, 38200 Vienne, France; 4 Laboratory Systemic Health Care, EA 4129, University of Lyon 1, Lyon, France; 5 Research On Healthcare Performance (RESHAPE), INSERM U1290, Université Claude Bernard Lyon 1, Lyon, France; 6 Centre Hospitalier du Forez, Montbrison, France; 7 REULIAN Network, Firminy, France; 8 RAMU Network, Aurillac, France; 9 Centre Hospitalier Henri Mondor, Aurillac, France; 10 Emergency Department and Clinical Research Unit, Medipole Hopital Mutualiste, Villeurbanne, France; 11 Centre Hospitalier Le Corbusier, Firminy, France; 12 Department of Emergency Medicine and SAMU, Hospital Edouard Herriot, Hospices Civils de Lyon, Lyon, France

**Keywords:** Emergency department, Typology, Factor analysis, Organisation, Territory

## Abstract

**Background:**

We hypothesized that monitoring the volume of activity and overall performance indicators is not sufficient to understand the underlying differences between emergency departments. We aimed to understand the underlying common characteristics of emergency departments and map their typology in order to propose adaptive solutions, that would take into account territorial specificities and manage existing resources.

**Methods:**

We applied a multifactorial analysis based on input data at three levels; 1) the health care available in the area surrounding the emergency departments, 2) the level of medical technicality of the hospitals and 3) the profile of emergency department visits.

**Results:**

We included 73 emergency departments in this study, representing 93.6% of the emergency departments in our region and seven groups were retained. The smallest group (*n* = 5) included both public and private structures with low volumes of activity. These medical structures were associated with the shortest length of stay and one of the lowest hospitalisation rates. The largest group (*n* = 21) included only public structures in peri-urban areas, which were associated with the highest rate of hospitalization in the region. The surrounding population was representative of the regional population, but the patients were older.

**Conclusions:**

This approach represents a systemic response to target the organisational needs and constraints, propose appropriate solutions and adjust the financial resources allocated to hospitals. Future policies to improve care delivery may benefit from stratifying solutions and performance objectives depending on these groups.

**Supplementary Information:**

The online version contains supplementary material available at 10.1186/s12874-021-01305-x.

## Background

Emergency departments (EDs) represent the main entrance to the hospital and provide rapid access to immediate life-saving assessment and treatment, regardless of the time and location. However, the ability of EDs to provide rapid and efficient care is far too often hampered by lack of capacity, particularly due to crowding [1, 2]. Initial causes are an increase in the number of visits for a decreasing number of beds, the inappropriate ED admissions of patients who could theoretically be addressed at the primary care [3] or ambulatory level [4] and access block relative to hospital operating capacity [5]. In addition, the delay in seeking care and treatment may account for some of the overall increase in the use of EDs which have also to handle the continued influx of elderly [6, 7] and chronic diseases [8]. These causes raise questions regarding the conditions of accessibility especially for out-of-hours consultations [9] and the provision of community-based care when urban agglomerations are associated with high medical density. In France, 8.1% of the population are currently facing a situation of poor accessibility with less than 2.5 consultations per inhabitant per year within 20 min of their home, taking into account the density of doctors and the health care needs of the inhabitants in their municipality and in neighbouring municipalities [10, 11]. This proportion will increase in the coming years, gradually spreading to new territories that have been spared so far, and accentuating ED crisis [12].

The increasing demand on EDs creates pressures on the health care system and may negatively influence the quality of care provided. Recent literature has generalized potential solutions regardless of available internal and external resources, by striving to achieve generalized performance objectives [13, 14]. For instance, the 4-h rule brings significant organisational improvements [15–17] but the identified success factor is a hospital-wide response, rather than an ED-centred approach [18]. With these experiences, we aimed to go further in identifying solutions for improving organisational models considering structural and environmental factors. We hypothesized that looking at volume of activity and overall performance indicators is not sufficient to understand the underlying differences between EDs, particularly since surrounding health care structures and patient accessibility differ from one territory to another, and from one surrounding population to another.

We aimed to understand the underlying common characteristics of EDs and map their typology in order to propose adaptive solutions that would take into account territorial specificities and manage existing resources. To do so, we applied a statistical multifactorial methodology without prior hypothesis, reproducible to other territories, based on an input data at three levels; the healthcare available in the surrounding the EDs, the level of medical technicality of the hospitals and the characteristics of ED visits. This approach represents a systemic response to target the organisational needs and constraints, propose appropriate solutions and adjust the financial resources allocated to hospitals.

## Method

### Data sources

We conducted an observational retrospective study based on the following open-source medico-administrative datasets collected over the year 2017. The regional observatory of EDs (ORU) participates in the collection and processing of emergency room summaries in order to improve quantitative and qualitative knowledge of the activity of emergency services, improve the conditions of patient management by these services and adjust the primary care offer to the needs of the population. In the Auvergne Rhône-Alpes (ARA) region in France, 77 EDs recorded more than 2.3 million ED visits per year, representing 11% of ED visits in France [19]. The National Institute of Statistics and Economic Studies (INSEE) is responsible for the production, analysis and publication of official statistics in France and provides quantitative data about the demography and the economy at the level of the territory. The National File of Health and Social Establishments (FINESS) indexes all French hospitals and clinics, retirement homes, pharmacies, biological laboratories or radiology offices. The Institute for Research and Documentation in Health Economics (IRDES) produces statistical data and analyses on the French health care system. Finally, the regional observatory for emergencies and the direction of Research, Studies, Evaluation and Statistics (DREES) produces statistics and socio-economic studies, including annual statistics by institution.

### Definition of catchment area

With the ED as statistical unit, the medico-administrative databases have to be linked and organised to describe each ED. This step implied to define a catchment area per ED, reflecting its geographical attractiveness. We defined the catchment area of an ED as the most represented residential places of the patients admitted to this ED. So, for each ED, we extracted all the zip codes of residential places of admitted patients from the ORU database. Then, the mean number of visits per zip codes was considered as a threshold to define a catchment area. Consequently, the zip codes with frequencies above this threshold were selected in the catchment area. We estimated that the mean was as relevant statistical parameter due to its sensitiveness to low and high values. Furthermore, common zip codes may be associated to several EDs because they reflect the real-life conditions where patients living in the same place could visit different ED.

### Data selection

The first level represented the territory of the hospital. The organisational density indicators corresponded to quantitative datasets extracted from the INSEE database such as the number of inhabitants, distribution of inhabitants by age and sex, distribution of inhabitants by socio-professional categories (farmers, craftsmen tradesmen and company managers, executives and higher intellectual professions, intermediate professions, employees, workers, retirees or without professional activity), number of general practitioners and nurses per inhabitants; from the FINESS database such as the number of housing project for dependant elderly people and the number of beds; from the IRDES database such as the localized potential accessibility to general practitioners (LPA indicator) and from the Corine Land Cover inventory such as the land use dichotomized in urban or rural if at least 50% of the territory is rural.

The second level represented the medical technicality of the hospital using qualitative variables. The status of the hospital (private/public), the level of medical technicality (presence or absence of a triage nurse on admission, the number of hospital beds and the number of full-time equivalent (FTE) physicians or nurses at the ED, the proportion of long- and short-term hospitalisation and the number of Emergency Mobile Services (EMS) transports were extracted from the ORU and the DREES database. We extracted the number of admissions and length of stay in ER from the ORU, the presence or absence of percutaneous coronary intervention center, stroke unit, resuscitation unit, intensive care unit, psychiatric services, and emergency medical service from the regional health agency (ARS).

The third level represented patient characteristics with data extracted from the ORU such as the distribution of patients by age and sex, the proportion of hospitalisations consecutive to the ED visit, and the severity using the French clinical classification of patients in the emergency department (CCMU) which evaluates the patient's condition in the emergency department, their level of clinical severity, and their medical prognosis (from 1—stable clinical conditions to 5 – life-threatening).

### Method selection

The high dimensionality and collinearity between these three input levels make standard regression techniques prone to unstable results and difficulties in generalization. Among the mathematical techniques for describing the underlying patterns in complex and correlated data sets, we choose the multiple factor analysis (MFA). This method allowed us to take advantage of the multi-level (territory, ED, patient) structure [20]. MFA is part of a family of multivariate statistical methods that aimed to identify the combination of variables that explains the greatest variability in a dataset. The most important variables are then reorganized into new synthetic variables, called axes, which are a linear combination of the original input variables. These new synthetic variables are numeric, centred around zero. Each individual can be represented as a point in this new space given its coordinates. This approach retains as much of the information in the dataset as possible while retaining only a subset of the synthetic variables. MFA has the particularity of organising variables and statistical units into groups and the advantage is to ensure that no single level can dominate the analysis [21].

### Data management

We provided imputations on missing values when these did not exceed the 20% threshold. Above this threshold, the proportion of missing data was considered too large to cover the missing values, and the decision was made to exclude these variables from the study. We also excluded 4 EDs with too much missing data. The Multiple Imputation by Chained Equations (MICE) method, based on a Monte-Carlo Markov Chain algorithm, was used to generate an imputed dataset. We quantified the territorial and demographic data from the raw data and produced ratios. For example, the number of general practitioners relative to the population residing in the health zone.

A constraint of MFA is to have data of the same type within each level. Some of the medical technicality level data were categorical, so we created categorical ordinal data for characterizing the structure’s medical technicality into groups. For example, the number of visits per structure was divided down into 4 classes; from 0 to 15,000, from 15,000 to 30,000, from 30,000 to 45,000, then above 45,000. When no a priori classifications were available, the continuous variables were divided into 5 classes using the “arules” package cluster method. After imputations, we retained 73 EDs in this study (93.6% of all EDs).

### Optimal number of axes

**Fig. 1:**
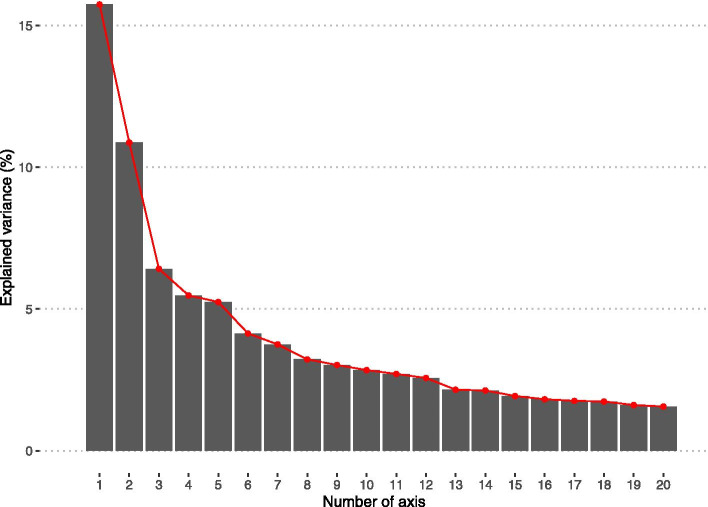
Part of explained variance per axis

After performing the mathematical transformations, we represented the variances explained from 20 first axes to select the first n axis that most capture the input data by minimizing the loss of information. To select the subset of axes to keep in the analyses, the elbow method was used. We observed a clear decrease in eigenvalues between the 3rd and 4th axis (Fig. 1) and a second one between 5 and 6^th^ axis. We decided to keep only first three axes, representing 33.04% of the total variance explained. Retaining a limited number of axes makes it possible to keep interpretability of each axis and to keep only the major characteristics in the analysis while removing local characteristics. This approach guarantees a better generalisation of the results to other regions. The 4th and 5th axis both explained 5% of the total variance and were uninterpretable.

### Optimal number of clusters

**Fig. 2:**
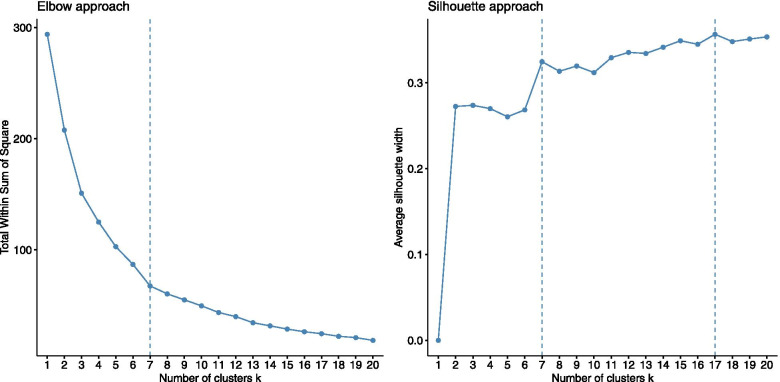
Optimal number of clusters according to the elbow approach on the total within sum of square and the average silhouette width

From the ED coordinates obtained after MFA, we used two methods to establish the optimal number of ED groups. The first method was based on the silhouette coefficient which reflects the quality of the clustering. The higher the value, the better the clustering. The second method consists in looking for the point on the elbow at which the reduction in variance obtained by adding a cluster reaches a point of diminishing returns. Both methods indicated 7 clusters as a satisfactory number (Fig. 2). From this result, the Partitioning around medoids (PAM) algorithm [22] was used to create the 7 clusters.

### Statistical analysis

We provided a description of the ED groups using the median and interquartile range of input at three levels as well as frequency and percentages. Statistical analyses were performed using R 3.6.1 software with the “*mice*” package for imputations [23], “*FactoMineR*” for running MFA methodology [24], “*factoextra*” for cluster selection [25], “*arules*” for discretizing variables [26] and “cluster” for kmedoid clustering [27].

## Results

### Contributions of variables to axes

**Fig. 3:**
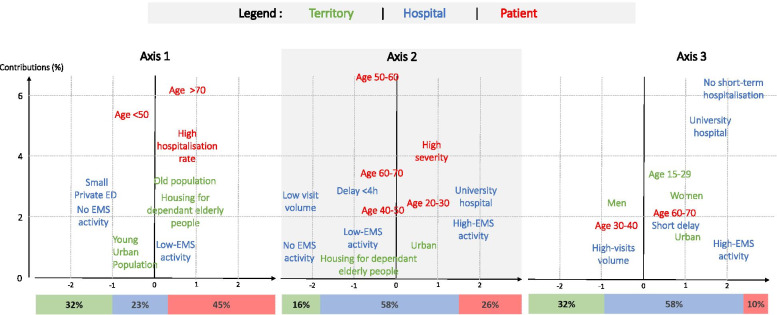
Contributions of the three-levels inputs to the first three axes

With the level contributions, we identified the variables with dominant effects per constituted axes (Fig. 3).

The first axis was made up of 45% of the patient variables, 32% of the territory and 23% of the hospital. Concerning the patient level, this axis discriminated between younger patients with negative coordinates and older patients with higher hospitalisation rates with positive coordinates. In terms of area level, this axis discriminated between younger, more populated and urban areas with negative coordinates and older areas with more housing project for dependant elderly with positive coordinates. Regarding the hospital level, this axis discriminated between small private hospitals with negative coordinates and public hospitals with positive coordinates.

For the second axis, the hospital level and patient level have the most important discriminating role in the axis with contributions about 58% and 26%. This axis discriminated an activity of high technicality/volume, long management delay and an important EMS activity in positive coordinates and an activity of low technicality with few beds per shift in negative coordinates.

The contribution to the third axis was approximately 58% for the hospital level and 32% for the territory level.

### Main characteristics

**Fig. 4:**
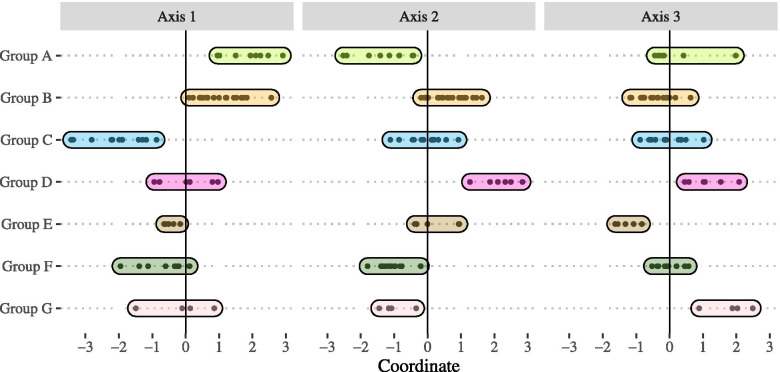
2D-mapping of the overlapping clusters per axis

The seven groups are described in terms of key characteristics per axis (Fig. 4) (See additional file).

The nine EDs in the group A were located in a rural residential area with 104,633 inhabitants [72,413;159,168] represented by 52.03% of residents older than 44 years old [49.19%;55.67%], and 31.27% older than 60 years old [28.40%;34.00%]. The most represented socio-professional category was “retired” with 34.16% of residents [31.61%;35.85%]. Associated with low medical technical specificities (1 intensive care unit, 1 coronary angiography suite, 2 psychiatry departments), the 9 EDs were public hospitals with low activity (12,010 visits per year [9,726;13,605]) associated with low referrals to EMS (519 EMS per year [479;801]). This group was also associated with the highest proportion of ED visits of less than 4 h (81.49% [78.45%;90.24%]) although only 4/9 hospitals have an ED triage nurse. The visits concerned patients with a stable clinical condition (82.48% [62.86%;87.04%]). We also observed the lowest accessibility to general practitioners over these territories (38.93 LPA [38.55;44.88]).

Group B involved 21 public hospital EDs including one teaching hospital with specialized technical care (13 intensive care units, 8 coronary angiography suites, 12 neurovascular units, 4 psychology units). Located in peri-urban areas, the surrounding population was representative of the regional population with a similar age distribution. The patient population was older, with 50.65% of patients older than 50 years old [47.69%;51.52%], and 26.44% of patients older than 70 years old [24.53%;27.40%]. These EDs were associated with the highest rate of hospitalization in the region with 26.61% [22.67%;29.83%].

With highly urbanized areas and high population densities (615,129 inhabitants [510,187;976,622]), group C included 13 EDs, with 11 private and 2 public hospitals. The 15–45 years old inhabitants were over-represented (42.98% [38.65%;44.08%]) compared to the regional population (36.09% [34.20%;40.08%]). In addition, the number of consultations by general practitioners was one of the highest in the region, at 51.2% [47.59%;57.36%]). These 13 EDs had a distinctive organization with the lowest physician FTE per 1,000 visits (0.28 [0.27;0.35]) and the lowest nurse FTE per 1,000 visits (0.61 [0.53;0.71]), without EMS.

Seven EDs formed group D, including 5 teaching hospitals and 2 public hospitals. Located in highly urbanised areas (0.23 [0.06;0.39]), the area is associated with a large supply of urban medical care (55.64% [50.39%;58.21%]) and few housing units for dependant elderly (0.84 [0.83;0.9]) per 10,000 inhabitants. These establishments were specialised technical medical structures (6 intensive care units, 5 coronary angiography suites, 2 neurovascular units, 2 psychology units), numerous EMS dispatch (4,210 [725;5,445]), a large number of beds (18.32 per 1,000 visits [10.35;21.03]), the highest bed occupancy rate in short-term hospitalization unit (235% [213%;331%]) and among the highest hospitalization rate (23.85% [22.04%;28.41%]). We also observed the highest nursing FTE per 1,000 ED visits with 1.11 [1.05;1.41]. The visits were associated with low severity situations (20.44% [18.77%;22.9%]) compared with the patients managed in the region (12.41% [7.16%;18.36%]).

Group E differed from group D in having a lower short-term hospital bed occupancy rates (122% [86%;154%]), fewer EMS dispatches (1,103 [870;1,639]) than the other groups and less management of life-threatening patients (0.22 [0.13;0.24]). This group included 5 public and 1 private hospital and had 29,944 [21,041; 33,591] visits per year.

With 6 public and 6 private hospitals, group F included hospital with low activity, less specialisation services (1 coronary angiography suite, 1 intensive care unit) without EMS. Localized in less urbanised areas, the hospitalisation rate was one of the lowest with 14.55% [9.39%;19.72%] mainly due to stable patients (82.61% [80.32%;84.97%]).

Group G included 4 public hospitals and 1 teaching hospital specialised in traumatology, with low activity (6,682 visits per year [6,225;11,293]). These medical structures were associated with the shortest throughput time (1.87 h [1.73;2.27]) and among the lowest hospitalisation rate (17.66% [14.01%;24.22%]).

**Fig. 5:**
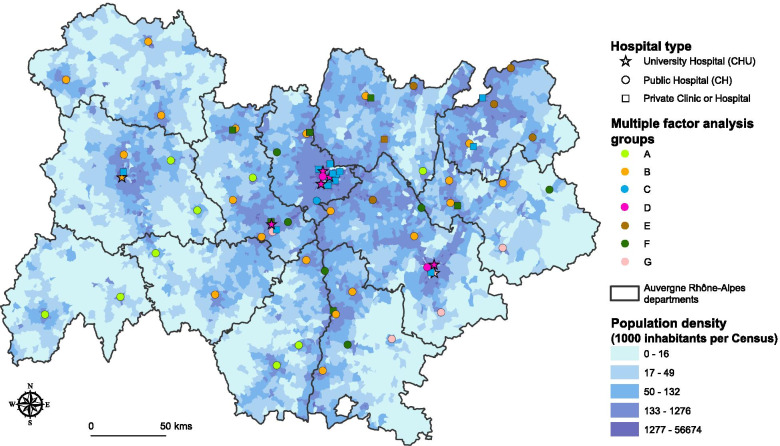
Spatial repartition of the groups of emergency departments

Spatial distribution of these groups is depicted in Fig. 5.

## Discussion

We proposed an automated and reproducible multi-level approach to understand the underlying typology of EDs in our region. Based on input variables at three levels, this approach considered 1) the territories and the surrounding health care supply, 2) the profile of patients admitted to ED and 3) the hospital activity with its technical specificities. One of its particularities is to not take into account any prior hypothesis.

The MFA methodology has been used and published in different search fields [21, 28, 29]. But the objectives were identical: to describe patterns in order to establish typologies. These applications illustrate that this approach does not only belong to the statistics but consists in extracting knowledge from structured datasets.

In this study, the low variance explained and evaluated on the first three axes (about 33.04%) highlighted the heterogeneity of ED characteristics and confirmed the relevance of the three-level approach in understanding the EDs typology. With more than three axes, the interpretation would consequently have been subject to local specifications that could not necessarily be generalized. The clustering methodology isolated 7 groups. Their main characteristics highlighted a great heterogeneity in the partitioning of EDs because the interpretability of the groups formed was in the same direction with no structure standing out from the others and no outliers. Moreover, beyond our preconceived ideas, the resulting partition of the EDs no longer stopped at a dichotomy between private and public status as in group C, E and F. For low or moderate severity, the territory and population levels were dominant; the principle of geographic proximity prevails for the choice of the primary care ED. But when the situation is more serious, patients are admitted to EDs of teaching hospital, regardless of where they live or where the accident occurred.

This methodological approach provided 7 distinct groups of EDs in the Auvergne Rhone-Alpes region. These groups are associated with different characteristics depending of the geography of the territory, the technical capabilities of its hospitals and its population. Furthermore, we observed that these 7 categories of EDs are funded in the same way, yet they behave differently. It would probably be necessary to adapt the financing of its structures, and to move towards a territorial and population-based logic. If we want to carry out a reform of the French health system in favour of the ED, we realize that a single reform would not be appropriate and that it would probably take 7 measures to meet the functional needs over the territory.

## Conclusions

These exploratory results showed that generalized approaches are a promising methodological tool to be explored, tested and improved, and confirmed the existence of an underlying EDs typology that provides an avenue for developing optimized organisational solutions. A future task force will therefore be interested in the performance of these structures and verify whether there is also a typology of performance. Future policies aimed at improving the care delivery, might benefit from stratifying solutions and performance objectives depending on these ED groups.

## Supplementary Information

**Additional file 1.** Main characteristics of the 7 groups. Median and interquartile range for continuous variables. Frequencies and percentages for categorical variables.

## Data Availability

Data generated or analysed during this study are included in this published article and open-source available, except the ORU data which are not publicly available due to institutional properties, but are available from the corresponding author on reasonable request.
