# Supplementary material for: Discovering the underlying typology of emergency departments
Source: BMC Med Res Methodol. 2021 Jun 5;21:116. doi: 10.1186/s12874-021-01305-x (PMC8180120; doi:10.1186/s12874-021-01305-x)
Supplement: Supplementary file 1 — Additional file 1. Main characteristics of the 7 groups. Median and interquartile range for continuous variables. Frequencies and percentages for categorical variables. [file 12874_2021_1305_MOESM1_ESM.docx]

Supplemental material: Main characteristics of the 7 groups.

Median and interquartile range for continuous variables. Frequencies and percentages for categorical variables.

|  | Group A  n=9 | Group B  n=21 | Group C  n=13 | Group D  n=7 | Group E  n=6 | Group F  n=12 | Group G  n=5 |
| --- | --- | --- | --- | --- | --- | --- | --- |
| Level 1 - Territory | | | | | | | |
| Age of inhabitants | | | | | | | |
| - 0-14 years old | 16.9 [14.6;18.15] | 18.3 [17.5;18.8] | 18.8 [18.3;19.5] | 18.4 [18.3;18.7] | 19.9 [19.6;20.2] | 18.5 [18.3;19.9] | 16.4 [16.2;17.9] |
| - 15-29 years old | 14.1 [13.1;14.5] | 16.3 [15.1;18.1] | 22.4 [17.8;23.5] | 22.2 [20.5;23.1] | 17.3 [16.6;17.6] | 16.6 [15.8;18.2] | 24.3 [20.9;25.2] |
| - 30-44 years old | 17.1 [16.6;17.9] | 18.5 [17.7;19.2] | 20.6 [19.1;20.6] | 19.9 [18.9;20.1] | 20.7 [20.0;21.9] | 18.8 [18.2;19.3] | 19.2 [18.9;19.4] |
| - 45-59 years old | 21.2 [20.7;21.6] | 20.3 [19.6;20.6] | 17.7 [17.1;19.7] | 17.5 [17.2;18.9] | 19.7 [19.6;20.5] | 19.9 [19.4;20.4] | 16.9 [16.2;18.8] |
| - 60-74 years old | 19.4 [17.7;20.2] | 16.8 [15.6;17.5] | 12.7 [12.3;14.6] | 13.1 [12.8;14.7] | 14.3 [13.6;14.6] | 15.9 [15.4;16.5] | 13.7 [13.7;14.6] |
| - 75-89 years old | 10.3  [9.2;11.9] | 8.6  [7.9;9.5] | 6.8  [6.5;7.4] | 7.3  [7.1;7.7] | 6.5  [6.3;6.9] | 8.2  [7.7;8.9] | 8.0  [7.7;8.2] |
| - >89 years old | 1.6  [1.4;1.8] | 1.3  [1.2;1.5] | 0.9  [0.9;1.0] | 1.1  [1.0;1.1] | 0.9  [0.8;0.9] | 1.2  [1.1;1.3] | 1.3  [1.2;1.3] |
| - Proportion of men | 48.4  [48.3;48.9] | 48.5  [48.4;48.9] | 48.3  [48.1;48.8] | 47.9  [47.8;48.6] | 49.3  [49.0;49.5] | 48.7  [48.3;49.1] | 48.4  [48.2;48.5] |
| Socio-professional categories | | | | | | | |
| - Farmers | 1.3  [1.2;3.0] | 0.8  [0.6;1.2] | 0.2  [0.1;0.4] | 0.2  [0.1;0.3] | 0.4  [0.4;0.5] | 0.7  [0.5;1.0] | 0.2  [0.2;0.4] |
| - Craftsmen tradesmen and company managers | 4.2  [3.8;4.4] | 3.9  [3.5;4.2] | 3.3  [2.9;3.4] | 3.2  [3.1;3.3] | 3.8  [3.6;4.4] | 3.9  [3.6;4.4] | 2.9  [2.9;3.2] |
| - Executives and higher intellectual professions | 4.9  [4.2;5.4] | 6.8  [5.9;8.2] | 11.9  [10.2;13.9] | 13.4  [12.7;13.7] | 7.8  [6.9;9.2] | 7.4  [6.8;8.2] | 13.3  [12.3;13.7] |
| - Intermediate professions | 11.6 [10.8;13.4] | 14.3 [12.9;15.7] | 16.3 [15.8;16.7] | 15.8 [15.8;16.1] | 16.4 [16.1;16.8] | 14.9 [13.9;16.0] | 14.9 [14.5;15.0] |
| - Employees | 15.77 [14.84;15.79] | 15.57 [15.29;15.98] | 16.03 [15.24;16.44] | 15.34 [15.23;15.38] | 16.49 [16.28;18.1] | 15.5 [15.11;15.8] | 14.9 [14.6;15.2] |
| - Workers | 13.8 [12.9;16.1] | 13.7 [12.8;13.9] | 11.4 [10.0;12.5] | 9.8  [9.6;10.4] | 15.2 [14.2;17.9] | 13.2 [13.0;14.2] | 9.5  [9.4;10.4] |
| - Retirees | 34.2 [31.6;35.8] | 29.4 [27.1;30.7] | 21.5 [20.7;24.3] | 22.5 [21.9;24.7] | 23.0 [22.3;25.3] | 27.8 [27.2;29.4] | 23.5 [23.5;24.4] |
| - Without professional activity | 12.7 [12.1;13.9] | 14.5 [13.5;15.7] | 18.7 [15.2;20.2] | 19.4 [17.9;19.8] | 14.0 [13.5;14.6] | 14.4 [13.6;16.2] | 20.1 [18.2;21.3] |
| Number of housing units for dependant elderly / 10 000 inhabitants | 1.6  [1.5;2.4] | 1.4  [1.1;1.5] | 0.7  [0.7;0.9] | 0.8  [0.8;0.9] | 0.8  [0.7;1.0] | 1.2  [1.2;1.4] | 0.9  [0.8;0.9] |
| Number of beds in housing units for dependant elderly / 10 000 inhabitants | 127.3  [113.2;176.4] | 91.5  [76.6;111.8] | 52.1  [46.8;61.5] | 58.8  [55.9;61.1] | 46.1  [43.9;71.6] | 89.0  [70.6;105.5] | 49.2  [47.3;51.8] |
| Number of nurses / 10 000 inhabitants | 18.9  [17.3;20.8] | 16.0 [14.2;17.8] | 14.5 [13.7;16.9] | 15.3 [14.8;16.5] | 9.71  [8.3;11.9] | 16.2 [14.2;18.3] | 18.0  [16.5;18.4] |
| Number of general practitioners / 10 000 inhabitants | 9.4  [7.9;10.1] | 9.6  [8.8;10.2] | 9.7  [9.3;10.6] | 10.6  [10.3;10.8] | 8.5  [7.9;9.4] | 9.8  [8.6;10.5] | 11.4  [11.3;12.1] |
| Level 2 - Hospital | | | | | | | |
| Duration of visit (hours) | 2.9  [2.0;3.5] | 3.9  [3.1;4.3] | 2.0  [1.6;2.6] | 4.6  [3.6;4.8] | 3.5  [3.1;3.9] | 2.3  [2.1;2.6] | 1.9  [1.7;2.3] |
| Proportion of short-term hospitalization | 10.9  [8.3;12.2] | 11.2  [8.8;14.4] | 3.6  [1.7;8.0] | 17.3  [12.7;26.9] | 6.2  [5.0;7.6] | 7.8  [2.8;11.8] | 0  [0;9.1] |
| Intensive care unit | 1 (11.1%) | 13 (61.9%) | 3 (23.1%) | 6 (85.7%) | 3 (50.0%) | 1 (8.3%) | 1 (20.0%) |
| Cathlab | 1 (11.1%) | 8 (38.1%) | 4 (30.8%) | 5 (71.4%) | 0 (0%) | 1 (8.3%) | 0 (0%) |
| Neurovascular unit | 0 (0%) | 12 (57.1%) | 0 (0%) | 2 (28.6%) | 1 (16.7%) | 0 (0%) | 0 (0%) |
| Psychological unit | 2 (22.2%) | 4 (19.1%) | 0 (0%) | 2 (28.6%) | 2 (33.3%) | 0 (0%) | 0 (0%) |
| Presence of a triage nurse | 4 (44.4%) | 21 (100%) | 11 (84.6%) | 7 (100%) | 6 (100%) | 10 (83.3%) | 3 (60.0%) |
| Number of beds / 1000 visits | 7.7  [6.6;9.4] | 10.8  [8.9;12.6] | 7.9  [5.1;9.7] | 18.3  [10.3;21.0] | 8.5  [6.9;8.8] | 6.3  [4.9;8.8] | 8.4  [7.7;8.6] |
| Hospital status: |  |  |  |  |  |  |  |
| - Teaching hospital | 0 (0%) | 1 (4.76%) | 0 (0%) | 5 (71.43%) | 0 (0%) | 0 (0%) | 1 (20.0%) |
| - Private hospital | 0 (0%) | 0 (0%) | 11 (84.6%) | 0 (0%) | 1 (16.7%) | 1. (50.0%) | 0 (0%) |
| - Public hospital | 9 (100%) | 20 (95.2%) | 2 (15.4%) | 2 (28.6%) | 5 (83.3%) | 6 (50.0%) | 4 (80.0%) |
| Full-time equivalent of physician / 100 visits | 0.3  [0.2;0.4] | 0.4  [0.3;0.4] | 0.3  [0.3;0.34] | 0.5  [0.2;0.6] | 0.5  [0.4;0.6] | 0.4  [0.3;0.4] | 0.6  [0.4;0.8] |
| Full-time equivalent of nurses / 100 visits | 1.0  [0.6;1.1] | 0.9  [0.8;1.1  ] | 0.6  [0.5;0.7] | 1.1  [1.0;1.4] | 0.9  [0.8;1] | 0.7  [0.5;0.9] | 0.72  [0.6;1.1] |
| Level 3 - Patient | | | | | | | |
| Age of patients : |  |  |  |  |  |  |  |
| - 18-30 years old | 17.4  [16.7;18.5] | 21.3  [20.4;22.4] | 26.2  [25.3;29.1] | 23.9  [22.3;27.0] | 22.8  [22.5;23.6] | 22.5  [21.4;23.1] | 18.4  [17.5;20.7] |
| - 30-50 years old | 26.6  [25.2;27.8] | 28.9  [27;29.8] | 34.4  [33.1;35.7] | 30.2  [29.1;30.2] | 32.5  [31.9;33.7] | 30.9  [30.3;33.3] | 28.6  [27.3;28.7] |
| - 50-70 years old | 27.1  [26.5;28.5] | 23.8  [22.9;2.5] | 22.7  [21.8;24.43] | 22.4  [20.4;22.9] | 23.4  [22.9;23.5] | 26.6  [24.9;27.9] | 29.4  [26.3;29.6] |
| - 70-90 years old | 23.7  [23.1;24.3] | 21.7  [20.0;22.7] | 14.4  [12.3;15.7] | 20.1  [18.3;21.3] | 17.7  [17.1;18.0] | 18.3  [14.3;18.9] | 20.7  [20.5;21.0] |
| - > 90 years old | 4.9  [4.8;5.3] | 4.6  [4.2;5.5] | 1.7  [1.3;2.9] | 4.4  [3.5;4.8] | 3.3  [2.9;3.5] | 2.3  [1.8;3.6] | 3.5  [2.1;3.6] |
| - Proportion of men | 51.7  [51.4;52.3] | 50.5  [48.6;51.9] | 48.5  [48.3;49.8] | 51.4  [49.1;53.9] | 50.6  [50.1;50.7] | 52.1  [50.9;53.2] | 50.9  [50.3;50.9] |
| French clinical classification: | | | | | | | |
| - Clinical state considered stable | 15.6  [7.5;20.1] | 12.7  [8.3;19.1] | 7.7  [0.7;10.2] | 20.4  [18.8;22.9] | 12.2  [7.5;13.6] | 10.0  [6.5;14.9] | 8.3  [7.9;16.5] |
| - Stable lesion status and/or functional prognosis | 62.1  [45.9;78.5] | 60.3  [49.8;7] | 61.7  [60.2;77.8] | 59.2  [56.3;61.7] | 67.2  [66.3;71.9] | 68.7  [67.3;77.1] | 73.5  [59.5;91.4] |
| - Injury state and/or functional prognosis judged to be at risk of degradation | 11.8  [9.3;15.5] | 12.8  [9.9;19.9] | 10.9  [6.9;21.3] | 19.2  [13.0;21.2] | 12.9  [7.3;15.9] | 13.8  [11.5;17.1] | 8.2 [  0.8;19] |
| - Pathological situation engaging the vital prognosis | 0.6  [0.3;0.9] | 1.2  [0.8;2.1] | 0.5  [0.2;0.9] | 0.9  [0.9;1.4] | 0.8  [0.5;1.1] | 0.6  [0.2;0.7] | 1.1  [0.1;1.4] |
| - Life-threatening situation | 0.1  [0.1;0.2] | 0.3  [0.2;0.5] | 0.1  [0.0;0.1] | 0.4  [0.4;0.6] | 0.2  [0.1;0.2] | 0.04  [0.02;0.06] | 0.2  [0.2;0.3] |
| Hospitalization rate | 25.3  [22.6;32.5] | 26.6  [22.7;29.8] | 9.3  [6.4;11.3] | 23.9  [22.0;28.4] | 21.7  [21.1;23.2] | 14.6  [9.4;19.7] | 17.7  [14.0;24.2] |
